# COVID-19 and Acute Cholecystitis Management: A Systematic Review of Current Literature

**DOI:** 10.3389/fsurg.2022.871685

**Published:** 2022-04-12

**Authors:** Konstantinos Stavridis, Ioannis Liosis, Michael K. Konstantinidis, Georgios Kondylis, Argyrios Ioannidis

**Affiliations:** ^1^School of Medicine, National and Kapodistrian University of Athens, Athens, Greece; ^2^Department of General, Laparoscopic, Oncologic and Robotic Surgery, Athens Medical Center, Athens, Greece

**Keywords:** acute cholecystitis, COVID-19, antibiotics treatment, conservative treatment, percutaneous cholecystostomy

## Abstract

**Introduction:**

Since the beginning of the COVID-19 pandemic, many patients with clinically acute presentations have been approached differently. The fear of viral transmission along with the short period of study made patients delay their hospital visits and doctors reassess the approach of certain acute situations. This study aimed to assess the changes in the management of patients with acute cholecystitis before and during COVID-19.

**Methods:**

A systematic review of the literature using PubMed (MEDLINE), Scopus, and ScienceDirect databases was performed until 01 September 2021. Totally, two kinds of studies were included, those assessing the management of acute cholecystitis during COVID-19 and those comparing the periods before and during the pandemic. The outcomes recorded include management approaches, complications, and mean length of stay.

**Results:**

A number of 15 eligible articles were included in the study. During the pandemic, six studies revealed a shift toward conservative management of acute cholecystitis and five of them reported that conservative management was opted in 73% of the patients. On the contrary, data from all studies revealed that the surgical approach was preferred in only 29.2% of patients. Furthermore, when comparing the periods before vs. during COVID-19, the conservative approach was reported in 36.3 and 43.2% before vs. during COVID-19, respectively, whereas surgical intervention was performed in 62.5% of patients before COVID-19 and 55.3% during the pandemic. The length of stay was delayed when a non-surgical approach was selected in most studies. Complications, mainly classified by the Clavien-Dindo scale, were higher in the pandemic period.

**Conclusion:**

A tendency toward more conservative approaches was observed in most studies, reversing the previously used surgical approach in most cases of acute cholecystitis. In most of the examined cases during the COVID-19 pandemic, antibiotic treatment and percutaneous cholecystostomy were much more considered and even preferred.

## Introduction

Acute cholecystitis is an emergency condition, most commonly the result of gallbladder disease, and is usually presented with right upper abdominal pain, pain in the right shoulder, nausea, vomiting, and occasionally fever. During 2012, in the United States, it was the sixth most common gastrointestinal and pancreatic diagnosis from emergency department visits, accounting for a total of 651,829 emergency department visits and 389,180 hospital admissions, amounting to 0.7 per 100,000 mortality rate (1).

According to the World Emergency Surgery Association (WSES) (2) and the Tokyo Guidelines (3), early laparoscopic surgery is the gold standard and should be performed as soon as the diagnosis is made and the choledocholithiasis risk is evaluated. This approach results in a shorter length of stay (LOS), fewer complications in comparison to late cholecystectomy, and generally decreased recurrence rates. Patients who are at high risk of morbidity or mortality should undergo conservative treatment and in case of failure, percutaneous cholecystostomy (PC) could serve as an alternative (2, 3).

During 2019, a new coronavirus named SARS-CoV-2 was identified. The related disease had a great social and financial global impact and was soon recognized as a pandemic (4). During such times, the worldwide healthcare systems and the management of surgical interventions were compromised. Due to the increased rate of hospitalizations, many organizations amended their guidelines to limit admission rates, so that they could free up space for possible patients infected with COVID-19 and limit patient exposure in a heavily infected environment. Based on this, organizations such as the British Intercollegiate General Surgery Guidance (BIGSG) (5), the Society of American Gastrointestinal and Endoscopic Surgeons (SAGES) (6), and the European Association for Endoscopic Surgery (EAES) (7) have stated that a more conservative approach to surgery, which means antibiotic therapy, PC, and “watchful waiting,” is preferred, whenever possible, in acute cholecystitis (5–7). On the other hand, WSES highly suggests that laparoscopic cholecystectomy should remain the standard of care even in the setting of the COVID-19 pandemic and warns against the excessive use of PC (8).

The rationale for this study is that no systematic review currently examines the shift toward a more conservative approach in the management of acute cholecystitis and the related outcomes in the COVID-19 era. This research aims to assess the impact of COVID-19 on acute cholecystitis management and its treatment.

## Methods

A systematic review of the literature was performed. The studies evaluating the management of acute cholecystitis during the COVID-19 period, as well as those comparing COVID-19 and non-COVID-19 periods, were included. Case reports, case series, abstracts, congress proceedings, and non-English language reports were excluded from our review. Studies with less than 15 patients were also excluded. The outcome measures taken into consideration were the rates of different management strategies during both the pandemic period and as a comparison between COVID-19 and pre-COVID-19 period. The rates of complications and mean LOS during those periods were also examined.

### Literature Search

**Figure 1:**
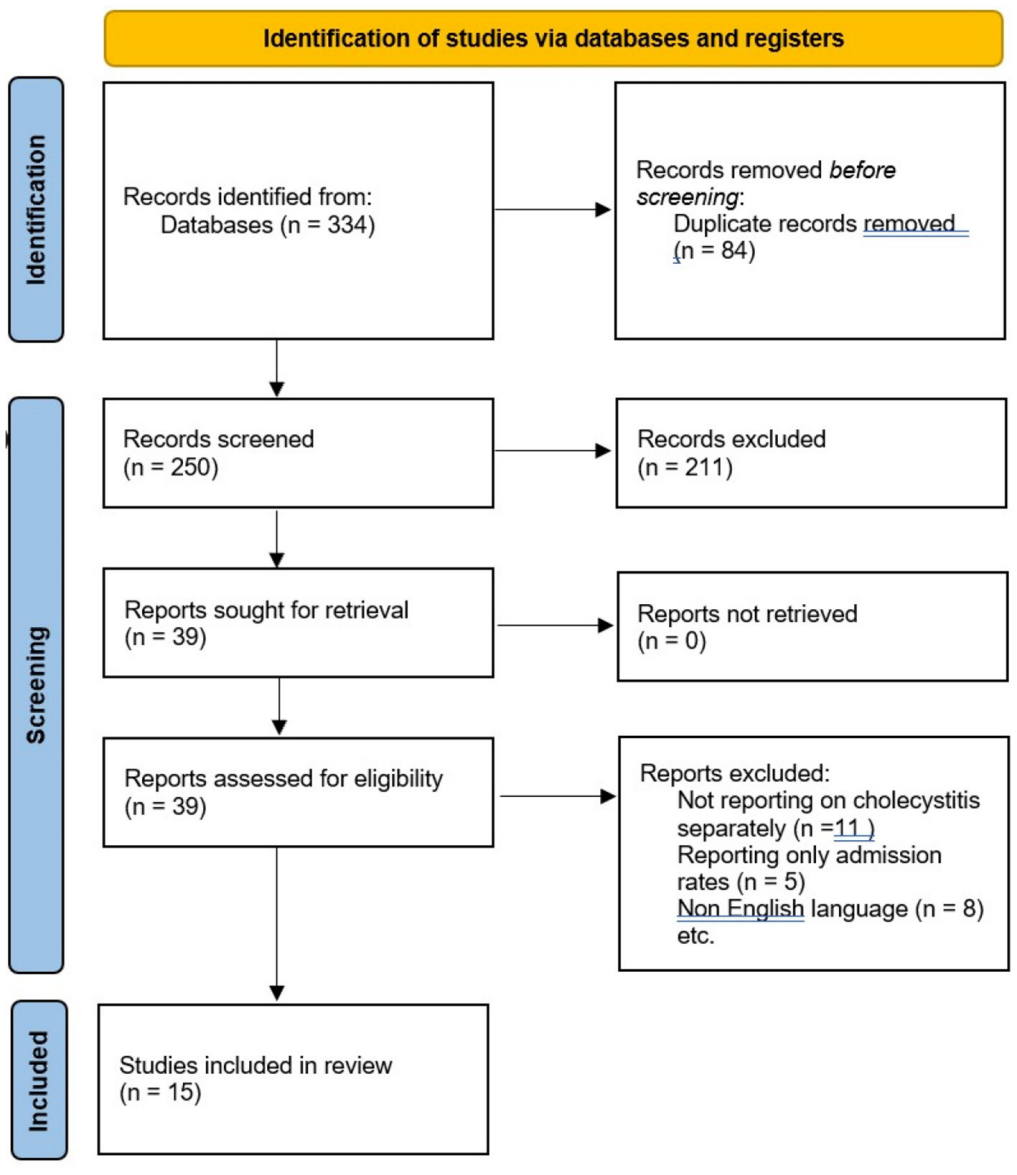
Preferred reporting items for systematic review and meta-analyses (PRISMA) 2020 flow diagram for study selection.

The two independent researchers (SK and LI) performed a literature search using PubMed (MEDLINE), Scopus, and ScienceDirect on 01 September 2021. The search terms used were “COVID-19” OR “SARS-CoV-2” AND “Acute Cholecystitis.” Review articles were hand-searched to identify any remaining studies. The preferred reporting items for systematic review and meta-analyses (PRISMA) guidelines (Figure 1) were followed (9). A registered review protocol was not used; however, the search strategy of one database (PubMed) is reported in the Supplementary Material.

### Data Collection and Analysis

The same two independent researchers screened titles and abstracts produced through our search strategy, and full texts of relevant articles were obtained. Eligibility was independently assessed by each author. One of the senior authors acted as a mediator whenever there was a disagreement between the two main reviewers with regards to the inclusion or exclusion of a paper. The quality of each study was assessed using the Oxford level of evidence (10). Data retrieved from each paper included the country and duration of the study, the type of study, the level of evidence, the patient number, the age, and the gender. Primary outcomes consisted of types of management of acute cholecystitis during the COVID-19 period and comparison of different treatments during the COVID-19 and pre-COVID-19 period. Secondary outcomes consisted of complication rates and mean LOS between those periods. Excel® (Microsoft, Redmond, WA, USA) was used for data handling and analysis. Each author was independent and blinded at the time of the data extraction.

## Results

### Search Results and Study Characteristics

A total of 334 potential articles were identified from the search of electronic databases. A total of 39 full-text articles were assessed for eligibility and 15 articles dated since 2019 were included in the study (11–25) (Figure 1). Three research studies were conducted in Italy, three in Turkey, two in Spain, one in the United Kingdom, one in New Zealand, one in the United States, one in Egypt, one in Austria, one in Switzerland, and one in Ireland. In terms of the study design, cohort studies and one survey reporting data regarding the three different approaches for acute cholecystitis were included (Table 1).

**Table 1:** Characteristics of different studies.

**References**	**Country**	**Duration**	**Multicenter**	**Type**	**N**	**Males**	**Age**	**Level of evidence (Oxford)**
Barabino et al. (12)	Italy	27 Feb-30 April 2020	No	Retrospective Cohort	37	21	64	2b
Martínez Caballero et al. (18)	Spain	01 March to 30th May 2020	Yes	Combined (Retrospective–Prospective) Cohort	257	146	69	2b
Shakir et al. (19)	UK	30 March 2020-26 April 2020	No	Retrospective Cohort	16	NA	56	2b
Hugo et al. (14)	Turkey	March 11 and May 31, 2020	No	Retrospective Cohort	72	32	57.3	2b
Perrone et al. (15)	Turkey	March 10 and June 10, 2020	No	Retrospective Cohort	36	17	68	2b
McGuinness et al. (22)	New Zealand	22 February to 25 2020 and 26 March to 27 April 2020	No	Retrospective Cohort	57	NA	NA	2b
Farber et al. (23)	USA	March and June, 2019- March and June 2020	No	Retrospective Cohort	53–80	55–68	46.7–48.8	2b
Fouad et al. (11)	Egypt	June 15, 2019 to March 15, 2020- March 16, 2020 to March 16, 2021	Yes	Prospective Cohort	458–311	118–103	40.2–41.1	2b
Kurihara et al. (24)	Italy	21 February to 3 April 2019, same 2020	Yes	Survey	468–376	NA	NA	N/A
Presl et al. (25)	Austria	01 March−15 April 2019, same 2020	Yes	Retrospective Cohort	33–20	NA	NA	2b
Surek et al. (13)	Turkey	14 March−15 May 2019, same 2020	No	Retrospective Cohort	55–29	NA	NA	2b
Hugo et al. (14)	Switzerland	15 March to 20 April 2019, same 2020	Yes	Retrospective Cohort	30–31	10–15	51–54	2b
Perrone et al. (15)	Italy	March and April 2019, same 2020	No	Retrospective Cohort	34–17	NA	NA	2b
Guadalajara et al. (16)	Spain	March 14th to May 2nd 2019, same 2020 and 2021	Yes	Retrospective Cohort	169–130–219	102–76–115	66–70–64	2b
Kamil et al. (17)	Ireland	1 March to 31 May, same 2020	No	Retrospective Cohort	33 22	NA	NA	2b

*NA, not applied; N, number of patients with acute cholecystitis*.

### Primary Outcomes

#### During COVID-19

In 6 studies, there were 475 patients in total that were diagnosed with acute cholecystitis. Among which, five of these assessed patients in a specific timeframe during the pandemic period, whereas one study differentiated patient management based on the lockdown and pre-lockdown period. Data reported from all three categories in between the studies indicated that the most commonly selected approach was the non-surgical one with a total of 160/218 patients (73%). This approach consisted of either conservative management and sole use of antibiotics for 127 (58%) patients or PC for 33 (15%) patients. Martínez Caballero et al. (18) did not report exact numbers on all three categories, but it clearly indicates a shift toward conservative management. On the other hand, surgery was the selected approach for 139/475 patients (29.2%). This arguably low percentage could be attributed to the result of the growing concern of clinicians on the risks of laparoscopic operations (Table 2).

**Table 2:** Management of acute cholecystitis during COVID-19.

**References**	**A.C number**	**Antibiotics**	**P.C**	**Surgery**
Barabino et al. (12)	37	11	8	18
Martínez Caballero et al. (18)	257	NA	NA	81
Shakir et al. (19)	16	16	0	0
Hugo et al. (14)	72	61	11	0
Somuncu et al. (21)	36	14	14	8
McGuinness et al. (22)	57	25	0	32
Total	475	NA	NA	139

*A.C, acute cholecystitis; P.C, percutaneous cholecystostomy; NA, not applied*.

#### Before vs. During COVID-19

The different decisions concerning the management of patients with acute cholecystitis during the COVID-19 era in comparison with the pre-COVID-19 period were a matter of discussion in 9 studies. A total of 1,333 patients were studied before and 1,235 after the onset of the pandemic. Four studies reported numbers in all three different approaches regarding acute cholecystitis management and are therefore examined together (13, 15, 16, 23). Combining the results of these studies, 344 patients were examined before and 497 during the COVID-19 period. In total, conservative management was the preferred option in 125 patients (36.3%) before and 215 (43.2%) during the COVID-19 era. Surgical management was reported in 215 patients (62.5%) before and 275 (55.3%) during the pandemic. One study showed a relative increase in the number of PCs performed during the COVID-19 period (23). These results indicate that throughout the pandemic, there has been a slide tendency toward conservative management, whereas the surgical approach is less considered compared to the pre-COVID-19 period. Three studies, which only presented surgical data concerning acute cholecystitis management, reported, in total, 107 patients previously managed operatively vs. 68 patients during the pandemic (14, 15, 25). Another study showed that there was a shift toward the initial surgical approach of the patients (11). During the pre-COVID-19 era, a total of 458 patients admitted with confirmed cholecystitis were managed surgically following an average of 2.21 days from clinical presentation, whereas during the COVID-19-era, a total of 389 admitted patients were initially managed conservatively with intravenous antibiotics followed by oral antibiotics and PC when required. Out of the 389 non-surgically managed patients, 311 (79.94%) failed to comply with these treatments and were on average operated within 16.74 days from clinical presentation. The results revealed that after the initial conservative management, the inflammatory status progressed and equally the severity score significantly worsened, thus increasing the difficulties and complications during the intraoperative and postoperative periods (11). Finally, another study indicated a shift toward conservative treatment. In detail, during the pandemic, there was an increase of 200% in the use of PC (*n* = 6 vs. *n* = 2) and a 30.7% decrease in cholecystectomies performed (*n* = 61 vs. *n* = 88) (24) (Table 3).

**Table 3:** Management of acute cholecystitis before vs. during COVID-19.

**References**	**A.C number Before vs. During**	**Antibiotics Before vs. During**	**P.C Before vs. During**	**Surgical Before vs. During**
Farber et al. (23)	53 vs. 80	4 vs. 12	4 vs. 7	45 vs. 61
Fouad et al. (11)	458 vs. 389	0 vs. NA	0 vs. NA	458 vs. 311
Kurihara et al. (24)	468 vs. 376	NA	2 vs. 6	88 vs. 61
Presl et al. (25)	33 vs. 20	0	0	33 vs. 20
Surek et al. (13)	55 vs. 29	38 vs. 24	0	17 vs. 5
Hugo et al. (14)	30 vs. 31	0	0	30 vs. 31
Perrone et al. (15)	34 vs. 17	0	0	34 vs. 17
Guadalajara et al. (16)	169 (2019) vs. 130 (2020) vs. 219 (2021)	54 (2019) vs. 89 (2020) vs. 69 (2021)	0	115 (2019) vs. 41 (2020) vs. 150 (2021)
Kamil et al. (17)	33 vs. 22	29 vs. 21	0	4 vs. 1

*A.C, acute cholecystitis; P.C, percutaneous cholecystostomy; NA, not applied*.

### Secondary Outcomes

#### During COVID-19

A total of six studies that presented data during the pandemic period were evaluated taking into account the mean LOS as an outcome of their strategy (Table 2). In one study, results regarding LOS were not clearly stated (19). McGuinness and Hsee (22) retrospectively performed a comparison of the mean LOS before and during the lockdown period and found no statistically different results between the two periods. Moreover, two studies compared the mean LOS between PC and either conservative or laparoscopic approaches. Ciyiltepe et al. (20) reported an increase in the mean LOS in patients who underwent PC (9.2 days) compared to patients who were managed conservatively (3.9 days). Somuncu et al. (21) compared the mean LOS between PC and laparoscopic approach and subsequently reported an increase of LOS in the PC group. In a third study, the researchers found a similar post-procedural mean LOS of 9 days in patients who underwent PC (12). Finally, Martínez Caballero et al. (18) after comparing the mean LOS between surgical and non-surgical approaches revealed a statistically significant increase in the mean LOS, when non-surgical management was applied (9.74 vs. 4.48, *p* = 0.001).

As far as complications are concerned, mainly based upon the Clavien-Dindo classification (26), there were no surprising data to report. In two of the studies, only one complication was observed in each of them. More specifically, in the study by Somuncu et al. (21) there was one (7.1%) mortality due to cardiac arrest in the group of patients who underwent PC. Moreover, in the study by Barabino et al. (12) one out of the 8 patients who underwent a cholecystostomy experienced an immediate complication (transient parietal bleeding) requiring conservative treatment (blood transfusion and intravenous infusion of tranexamic acid). The study by Martínez Caballero et al. (18) reported an overall postoperative complications rate of 26%, with the most frequent ones classified as Clavien–Dindo grade I (70.1%, *p* < 0.01), while severe complications (grades IV–V) were noticed in 14.9% of patients. Mortality rate was 1.3 and 3.2% (*p* = 0.075) in surgical and non-surgical treatment groups, respectively. Mortality after PD was significantly higher (15.1%, *p* = 0.001) compared to cholecystectomy (1.2%) and antibiotic therapy (2.4%).

#### Before vs. During COVID-19

A total of nine studies evaluating patients with acute cholecystitis before and during the pandemic period compared the results between the aforementioned periods. Out of all studies, six of them measured the mean LOS as a secondary outcome of their research, while in the rest, results regarding the mean LOS were not applied. In general, all studies reported an increase in the mean LOS during the COVID-19 period, which suggests an unsuccessful approach for the management of acute cholecystitis during the pandemic. Two studies found a statistically significant increase in the average LOS during the pandemic period compared to the pre-COVID-19 era (11, 15). Fouad et al. (11) reported the most significant difference in the average LOS between the two periods (13.5 days in 2020 vs. 2.6 days in 2019). It is of high importance to mention that the low average LOS of the pre-COVID-19 period is a result of a complete surgical strategy, whereas, in 2020, conservative management was also applied. Farber et al. (23) reported similar results regarding median hospital LOS in surgically managed patients between the two periods. Finally, Guadalajara et al. (16) found a prolonged LOS in 2020 (6 days) compared to 2019 and 2021 (4 days both). This observation can be explained by the selection of conservative treatment and the fewer laparoscopy rates during the first wave of the pandemic, which can be attributed to concerns about the transmission of the virus with aerosolization.

As far as the complications are concerned, the majority of studies presented the complication rates based on the Clavien-Dindo classification (Table 4). Three of the articles comparing COVID-19 and the pre-COVID-19 era did not clearly mention the complications of their management strategies (13, 24, 25). Perrone et al. (15) did not report any significant difference between the two periods, as only one death occurred in the COVID-19 era. Farber et al. (23) stated a slightly higher rate of complications during the COVID-19 period, which includes four cases of sepsis and one death, compared to the pre-COVID-19 period. Although not statistically significant, the researchers highlighted the existence of a longer duration of symptoms prior to presentation in the COVID-19 period, as a possible factor linked to this higher rate of complications. Other than that, Hugo et al. (14) reported more CDII complications in the pandemic period (7%) compared to 2019 (5%), as well as three complications of CDIII grade, whereas no CDIII complications are reported in the pre-COVID-19 era. Similarly, Kamil et al. (17) reported a higher rate of CDII complications in the pandemic period compared to the period before the viral spread. However, these differences are not statistically significant. Fouad et al. highlighted that the pandemic period was associated with the highest rate of postoperative complications, with 8.03% developing bile leakage, 5.14% having missed duct stones that needed further intervention with endoscopic retrograde cholangiopancreatography (ERCP), and 0.96% developed duodenal injury. Pulmonary complication rates were 6.11 and 19.6% before and during COVID-19, respectively (*p* < 0.05). These differences are also reflected in the Clavien-Dindo grading system, with 16.1% of patients presenting a CDIIIa or higher in the pandemic period. On the other hand, severe complications (CDIIIa or higher) were observed in only 0.6% of the population in the pre-COVID-19 era. The mild complication rate (CDI or CDII) was similarly higher in the pandemic period (11). Finally, Guadalajara et al. (16) reported increased complication rates of any Clavien-Dindo grade during 2020 compared both to 2019 and 2021. The difference between 2020 and 2021 is statistically significant (*p* = 0.026). However, no difference in the rate of severe complications (CDIII-IV) was observed between the 3 years.

**Table 4:** Complications before vs. during COVID-19.

**References**	**Before COVID-19**	**During COVID-19**
Perrone et al. (15)	0	1 death
Hugo et al. (14)	1 CDI (1%), 4 CDI (5%), 0 CDIII	1CDI (3%), 5CDII (7%), 2CDIII (3%)
Kurihara et al. (24)	NA	NA
Presl et al. (25)	NA	NA
Kamil et al. (17)	Inpatient 10 (16%), transaminitis 1 (2%)	Inpatient 11 (13%), 4 Sepsis (5%), 1 intra-abdominal abscess (1%), transaminitis 1 (1%), 1 death (1%)
Fouad et al. (11)	CDI (1.3%), CDII (6.3%), CDIIIa (0%), CDIIIb (0.21%), CDIVa (0.44%), CDIVb (0%)	CDI (11.6%), CDII (8.9%), CDIIIa (6.4%), CDIIIb (7.1%), CDIVa (2.6%), CDIVb (0%)
Guadalajara et al. (16)	Any CD grade: 28 (16.5%)	Any CD grade: 2020: 33 (25.2%) 2021: 33 (15%)
Kamil et al. (17)	1 CDII	1CDI, 3CDII
Surek et al. (13)	NA	NA

*CD, clavien-dindo classification; NA, not applied*.

## Discussion

During the COVID-19 era, there have been numerous modifications in the approach of emergent cases in every medical specialty and especially in situations with potential surgical intervention. Before the outbreak of this viral pandemic, laparoscopic cholecystectomy was the gold standard treatment in most patients diagnosed with acute cholecystitis. There are several studies in the current literature that suggest emergent surgery in acute cholecystitis, and the WSES further emphasizes that early laparoscopic cholecystectomy should be performed as soon as possible but can be safely performed up to 10 days after the onset of symptoms (2). However, early laparoscopic intervention is significantly associated with a shorter hospital stay, fewer complications, and operational costs (2, 27). Following the virus's global spread, guidelines regarding acute abdominal incidents were modified. Indeed, the BIGSG on COVID-19 stated that during the COVID-19 pandemic, whenever non-operative management is possible (such as for early appendicitis and acute cholecystitis), this should be performed. BIGSG recommended either non-surgical management or the utilization of a PC tube for the management of acute biliary disease (5). Similarly, other surgical societies, such as the SAGES and EAES, have also advocated for a more patient- and hospital-centered approach, which suggests conservative treatment whenever appropriate (6, 7).

With regard to the above, numerous hospitals considered altering their initial approach. This shift was mainly attributed to the fact that laparoscopy is an aerosol-forming procedure and carries a potential risk for transmission of SARS-CoV-2 to healthcare professionals (28). As a result, several hospitals began their treatment with antibiotics and “watchful waiting” while others performed PC. As a matter of fact, this potential risk was investigated and the results revealed a greater benefit in favor of laparoscopy with no reason of replacing it with laparotomy due to COVID-19 infection (28).

Additional safety precautions were recommended to avoid the possibility of virus transmission. Those measures concern mainly the prevention of pneumoperitoneum dispersion and potential viral spreading (29, 30), the safer operative technique with the proper evacuation of smoke developed from electrosurgery and ultrasonic surgery (29, 31), the disinfection of potentially contaminated devices and materials, and the usage of more protective equipment under each hospitals protocol. Finally, recommendations were proposed for the establishment of specific operating rooms for patients with COVID-19 regarding the risk of transmission between patients (29, 31, 32).

The various types of management of acute cholecystitis along with their outcomes were the topic of our systematic review. The studies were divided into two main categories with the first one focusing on different approaches exclusively during the COVID-19 outbreak, whereas in the second one, studies compared alternative managements before and after the start of the pandemic. In the first group, within a total of 218 patients with acute cholecystitis, there was a significantly high number of non-surgical treatments [160], of which 127 were only given antibiotics and 33 were treated with PC. In contrast, only a small number of patients [58] directly underwent surgery. The outcomes from most of the studies of the second category were similar. There was a notable change in the percentage of conservative management before (36.3%) and after (43.2%) the start of the pandemic. As a matter of fact, PC was frequently preferred on many occasions as a combination of potentially life-saving and less invasive treatment options, taking into consideration that it can serve as a bridge therapy allowing patients to survive severe disease and stabilize until they undergo a cholecystectomy (21). Moreover, in some cases, PC was chosen over surgery, taking into consideration the severity of pulmonary complications related to the disease (12, 33).

Treating patients conservatively as outpatients or inpatients does spare surgical capacity; however it renders the overall hospital stay much longer, and, in some cases, it reflects in more complicated cholecystitis. Our systematic review depicts that this is the result of both antibiotic therapy and PC. In the case of PC, the LOS was noted even longer which can be attributed to delay in PC insertion (20). In this setting, the WSES underlines that the extravagant use of PC jeopardizes the standard level of care and that this method should be reserved for only a small, selected subset of patients (8). The shift toward more conservative treatments was thought to minimize the risk of aerosol transmission of COVID-19 through laparoscopic procedures and, therefore, protect from the viral spread. However, this type of management is associated with extended hospitalizations and, therefore, longer viral exposure for the patients and the professionals. When comparing the two periods, several studies reported a higher rate of complications since the outbreak of COVID-19. More specifically, Fouad et al. (11) highlighted a statistically significant difference in intra-operative, post-operative, and non-surgical complications (predominantly pulmonary) during the pandemic compared to the pre-COVID-19 period. However, the longer LOS and complication rates during the COVID-19 period may be attributed to a prolonged duration of symptoms prior to admission, due to the patients' concern of possible virus transmission (11, 23).

The majority of studies were conducted during the onset of the pandemic, a period without straight facts about COVID-19. Nowadays, due to the contribution of many studies, knowledge has been acquired concerning both the transmission of the virus and the strategies that are necessary for the protection and risk minimization of healthcare providers. Therefore, it is a thought-provoking question whether the modifications in acute cholecystitis management analyzed in this review are still existent or whether treatment strategies have returned back to their prior state.

This systematic review has a number of limitations. First, our research was mainly based on retrospective studies with no available randomized trials, due to the ongoing status of the COVID-19 pandemic. Moreover, each study reports in different lockdown periods based on the country and the existing circumstances at the time of conduction, thus leading to a discrepancy in terms of quality and completeness of data between the studies. The fact that the observation period of the studies is not equally long should also be noted. In addition, this systematic review presents only the short-term outcomes of conservative treatment with no reference to the long-term recurrence rates of this approach. Finally, stratification of the results according to Tokyo grade could not be done as only a few studies used this classification.

In summary, the ongoing pandemic has had a tremendous impact on surgical emergencies, and thus, the management of acute cholecystitis could not pose an exception and has been dramatically affected. Most studies reported a tendency toward more conservative approaches, namely, the use of antibiotics or PC, for the treatment of acute cholecystitis, in comparison to the widely used early laparoscopic cholecystectomy in the pre-pandemic era. This review highlights that this approach is associated with a longer LOS and, in certain circumstances, higher complication rates. Due to the unknown course of the pandemic, future studies, especially randomized controlled trials, are compulsory to investigate the safety profile of non-surgical management for acute cholecystitis patients.

## Data Availability Statement

The original contributions presented in the study are included in the article/supplementary material, further inquiries can be directed to the corresponding author.

## Author Contributions

KS and IL: conceptualization. MK: methodology. GK, KS, IL, and MK: literature review, writing—original draft preparation, and project administration. AI: validation, writing, reviewing, editing, and supervision. KS and MK: formal analysis. IL and GK: investigation. KS: resources. IL: data curation. GK: visualization. All authors have read and agreed to the published version of the manuscript.

## Conflict of Interest

The authors declare that the research was conducted in the absence of any commercial or financial relationships that could be construed as a potential conflict of interest. The reviewer AM declared a shared affiliation with the authors KS, IL, and GK to the handling editor at the time of review.

## Publisher's Note

All claims expressed in this article are solely those of the authors and do not necessarily represent those of their affiliated organizations, or those of the publisher, the editors and the reviewers. Any product that may be evaluated in this article, or claim that may be made by its manufacturer, is not guaranteed or endorsed by the publisher.
