# Supplementary material for: COVID-19 and Acute Cholecystitis Management: A Systematic Review of Current Literature
Source: Front Surg. 2022 Apr 12;9:871685. doi: 10.3389/fsurg.2022.871685 (PMC9039201; doi:10.3389/fsurg.2022.871685)
Supplement: Supplementary file 1 [file Table_1.pdf]

Full - search strategy in Pubmed

| Search number | Query | Sort By | Filters | Search Details | Results |
|---|---|---|---|---|---|
| 4 | ((COVID-19) OR (SARS-CoV-2)) AND (acute cholecystitis) | Most Recent | | ("covid 19"[All Fields] OR "covid 19"[MeSH Terms] OR "covid 19 vaccines"[All Fields] OR "covid 19 vaccines"[MeSH Terms] OR "covid 19 serotherapy"[All Fields] OR "covid 19 serotherapy"[Supplementary Concept] OR "covid 19 nucleic acid testing"[All Fields] OR "covid 19 nucleic acid testing"[MeSH Terms] OR "covid 19 serological testing"[All Fields] OR "covid 19 serological testing"[MeSH Terms] OR "covid 19 testing"[All Fields] OR "covid 19 testing"[MeSH Terms] OR "sars cov 2"[All Fields] OR "sars cov 2"[MeSH Terms] OR "severe acute respiratory syndrome coronavirus 2"[All Fields] OR "ncov"[All Fields] OR "2019 ncov"[All Fields] OR (("coronavirus"[MeSH Terms] OR "coronavirus"[All Fields] OR "cov"[All Fields]) AND 2019/11/01:3000/12/31[Date - Publication]) OR ("sars cov 2"[MeSH Terms] OR "sars cov 2"[All Fields] OR "sars cov 2"[All Fields])) AND ("cholecystitis, acute"[MeSH Terms] OR ("cholecystitis"[All Fields] AND "acute"[All Fields]) OR "acute cholecystitis"[All Fields] OR ("acute"[All Fields] AND "cholecystitis"[All Fields])) | 77 |
| 3 | acute cholecystitis | Most Recent | | "cholecystitis, acute"[MeSH Terms] OR ("cholecystitis"[All Fields] AND "acute"[All Fields]) OR "acute cholecystitis"[All Fields] OR ("acute"[All Fields] AND "cholecystitis"[All Fields]) | 10,877 |
| 2 | SARS-CoV-2 | Most Recent | | "sars cov 2"[MeSH Terms] OR "sars cov 2"[All Fields] OR "sars cov 2"[All Fields] | 149,866 |
| 1 | COVID-19 | Most Recent | | "covid 19"[All Fields] OR "covid 19"[MeSH Terms] OR "covid 19 vaccines"[All Fields] OR "covid 19 vaccines"[MeSH Terms] OR "covid 19 serotherapy"[All Fields] OR "covid 19 serotherapy"[Supplementary Concept] OR "covid 19 nucleic acid testing"[All Fields] OR "covid 19 nucleic acid testing"[MeSH Terms] OR "covid 19 serological testing"[All Fields] OR "covid 19 serological testing"[MeSH Terms] OR "covid 19 testing"[All Fields] OR "covid 19 testing"[MeSH Terms] OR "sars cov 2"[All Fields] OR "sars cov 2"[MeSH Terms] OR "severe acute respiratory syndrome coronavirus 2"[All Fields] OR "ncov"[All Fields] OR "2019 ncov"[All Fields] OR (("coronavirus"[MeSH Terms] OR "coronavirus"[All Fields] OR "cov"[All Fields]) AND 2019/11/01:3000/12/31[Date - Publication]) | 235,101 |
